# Efficacy of vitamin D_3_ supplementation in reducing incidence of pulmonary tuberculosis and mortality among HIV-infected Tanzanian adults initiating antiretroviral therapy: study protocol for a randomized controlled trial

**DOI:** 10.1186/s13063-017-1819-5

**Published:** 2017-02-10

**Authors:** Christopher R. Sudfeld, Ferdinand Mugusi, Said Aboud, Tumaini J. Nagu, Molin Wang, Wafaie W. Fawzi

**Affiliations:** 1 Department of Global Health and Population, Harvard T.H. Chan School of Public Health, 665 Huntington Ave, Building I Room 1104C, Boston, MA 02115 USA; 2 Department of Epidemiology, Harvard T.H. Chan School of Public Health, Boston, MA USA; 3 Department of Nutrition, Harvard T.H. Chan School of Public Health, Boston, MA USA; 4 Department of Biostatistics, Harvard T.H. Chan School of Public Health, Boston, MA USA; 5 Management and Development for Health, Dar es Salaam, Tanzania; 6 Department of Internal Medicine, Muhimbili University of Health and Allied Sciences, Dar es Salaam, Tanzania; 7 Department of Microbiology and Immunology, Muhimbili University of Health and Allied Sciences, Dar es Salaam, Tanzania

**Keywords:** HIV, Tuberculosis, Vitamin D, Micronutrient, Cholecalciferol, Nutrition, Tanzania

## Abstract

**Background:**

HIV-infected adults initiating antiretroviral therapy (ART) in sub-Saharan Africa continue to experience high rates of morbidity and mortality during the initial months of treatment. Observational studies in high-income and resource-limited settings indicate that HIV-infected adults with low vitamin D levels may be at increased risk of mortality, HIV disease progression, and incidence of pulmonary tuberculosis (TB). As a result, vitamin D_3_ supplementation may improve survival and treatment outcomes for HIV-infected adults initiating ART.

**Methods/Design:**

The Trial of Vitamins-4 (ToV4) is an individually randomized, double-blind, placebo-controlled trial of vitamin D_3_ (cholecalciferol) supplementation conducted among 4000 HIV-infected adults with low vitamin D levels [25-hydroxyvitamin D (25(OH)D) <30 ng/mL] initiating ART in Dar es Salaam, Tanzania. The two primary aims of the trial are to determine the effect of a vitamin D_3_ supplementation regimen on incidence of (1) mortality and (2) pulmonary TB as compared to a matching placebo regimen. The primary safety outcome of the study is incident hypercalcemia. The investigational vitamin D_3_ regimen consists of oral supplements containing 50,000 IU vitamin D_3_ taken under direct observation at randomization and once a week for 3 weeks (four doses) followed by daily oral supplements containing 2000 IU vitamin D_3_ taken at home from the fourth week until trial discharge at 1 year post ART initiation. Trial participants are followed up at weekly clinic visits during the first month of ART and at monthly clinic visits thereafter until trial discharge at 1 year post ART initiation. Secondary aims of the trial are to examine the effect of the vitamin D_3_ regimen on CD4 T cell reconstitution, incidence of non-TB comorbidities, body mass index (BMI), depression and anxiety, physical activity, bone health, and immunologic biomarkers.

**Discussion:**

The ToV4 will provide causal evidence on the effect of vitamin D_3_ supplementation on incidence of pulmonary TB and mortality among HIV-infected Tanzanian adults initiating ART. The trial will also give insight to whether vitamin D_3_ supplementation trials for the prevention of pulmonary TB should be pursued in HIV-uninfected populations.

**Trial registration:**

ClinicalTrials.gov, NCT01798680. Registered on 21 February 2013.

**Electronic supplementary material:**

The online version of this article (doi:10.1186/s13063-017-1819-5) contains supplementary material, which is available to authorized users.

## Background

Access to antiretroviral therapy (ART) coverage has rapidly increased in sub-Saharan Africa during the last decade with over 12 million human immunodeficiency virus (HIV)-infected individuals on ART in 2015 [1]. In contrast to the success in expanding ART access, HIV-infected adults initiating ART in sub-Saharan Africa continue to experience significantly higher rates of morbidity and mortality during the initial months of treatment as compared to HIV-infected individuals in high-income settings [2, 3]. The 1-year mortality rate for HIV-infected individuals initiating ART in the urban Dar es Salaam, Tanzania HIV care and treatment program is estimated to be 13.1% [4]. As a result, low-cost interventions are needed to prolong and improve quality of life for HIV-infected individuals receiving ART in Tanzania and similar resource-limited settings.

Vitamin D is a potent immunomodulator with effects on both adaptive and innate immune responses which may play a prominent role in control of intracellular pathogens by enhancing cell-mediated immunity, production of antimicrobial peptides, and phagocytic activity of macrophages [5–7]. Accordingly, HIV-infected individuals with adequate levels of vitamin D may better control HIV replication and intracellular opportunistic infections as compared to individuals with suboptimal vitamin D [8]. Observational cohort studies have examined the association of vitamin D status with HIV-related morbidity and mortality in both high-income and resource-limited settings [9–17]. The largest study to date in high-income settings was conducted among a sub-sample of 1985 individuals enrolled in the EuroSIDA cohort, which consists of HIV-infected adults from 31 European countries, Israel, and Argentina [11]. The EuroSIDA vitamin D study determined individuals in the highest (>20 ng/ml) and middle (12–20 ng/mL) tertiles of 25-hydroxyvitamin D (25(OH)D) had 44% and 32% reduced risk of death as compared with individuals in the lowest tertile (<12 ng/mL), respectively [11]. To the best of our knowledge, there have been three prospective cohort studies of vitamin D conducted among HIV-infected adults in resource-limited settings [13–16]. The first vitamin D cohort study was conducted by our group among 884 HIV-infected Tanzanian pregnant women prior to availability of ART and we determined women in the highest quintile of 25(OH)D had 42% lower risk of all-cause mortality as compared with those in the lowest quintile [13]. In addition, <32 ng/mL 25(OH)D was associated with increased risk of progression to World Health Organization (WHO) HIV stage 3 or 4 disease and incidence of severe anemia, wasting, acute upper respiratory infections, and oral thrush [13]. We also more recently conducted a prospective cohort study of vitamin D among HIV-infected adult men and women initiating ART in Dar es Salaam, Tanzania [15, 16]. In this study we found individuals with vitamin D deficiency at ART initiation (25(OH)D levels <20 ng/mL) had twice the risk of mortality during the first 2 years of treatment as compared to individuals with vitamin D sufficiency (25(OH)D levels >30 ng/mL) [16]. HIV-infected adults initiating ART with vitamin D sufficiency also had reduced risk of incident pulmonary tuberculosis (TB), oral thrush, wasting, and >10% weight loss [15]. The third observational cohort study was conducted among 398 HIV-infected Ugandan adults receiving ART, which found individuals with vitamin D deficiency (<20 ng/mL 25(OH)D) had on average 65 cells/μL lower CD4 T cell counts at 18 months of follow-up as compared to individuals with vitamin D sufficiency (>30 ng/mL 25(OH)D). Overall, the observational evidence suggest HIV-infected individuals with low levels of vitamin D may be at increased risk of mortality, pulmonary TB, HIV disease progression, have poorer CD4 T cell reconstitution when on ART. Nevertheless, there are no published randomized controlled trials of vitamin D supplementation among HIV-infected adults in resource-limited settings published to date to determine causal effects.

The potential role of vitamin D in pulmonary TB immune responses has long been acknowledged as over 25 years ago Rook and colleagues published results showing the active form of vitamin D suppressed proliferation of *Mycobacterium tuberculosis* (Mtb) [18]*.* Over the past few decades, a large number of studies have examined the role of vitamin D in immune responses, particularly in macrophage function [6]. A study by Liu and colleagues found that ligation of macrophage toll-like receptor 2/1 by Mtb antigen induces expression of the vitamin D receptor and synthesis of 1,25(OH)_2_D [19]. Synthesis of 1,25(OH)_2_D by macrophages can then upregulate expression of cathelicidin, which is an antimicrobial peptide integral for killing Mtb and other intracellular pathogens [20, 21]. In addition, a landmark study by Fabri and colleagues determined vitamin D is required for an interferon-gamma (IFN-γ)-mediated antimicrobial pathway in macrophages which leads to autophagy, phagosomal maturation, and other antimicrobial activities against Mtb [5]. Generally, there is strong evidence from immunological studies suggesting that maintaining adequate levels of vitamin D may improve immune responses to TB.

There have also been a similarly large number of epidemiologic studies conducted on the relationship of vitamin D and incidence of pulmonary TB. Numerous case-control studies have determined individuals with active TB have significantly lower 25(OH)D and different genetic variants of the vitamin D receptor (VDR) as compared to healthy controls or the general population [22–26]. A recent case-control study among HIV-uninfected and HIV-infected South Africans found TB cases had about five times the odds of vitamin D deficiency (25(OH)D <20 ng/mL) as compared to population controls [27]. Nevertheless, these cross-sectional studies are prone to bias due to reverse causation since increased immune activation in individuals with active TB may lead to decreased 25(OH)D levels [6]. Only a few prospective cohort studies, which limit the potential of reverse causation, have examined the association of vitamin D and incidence of TB [15, 28, 29]. In one small prospective cohort study conducted in Pakistan, household TB contacts in the lowest tertile 25(OH)D (<7 ng/mL) had increased risk of developing TB, although these results are based on only eight incident TB cases [29]. Another small cohort study of TB contacts in Spain determined contacts with low vitamin D (<10 ng/mL 25(OH)D) had increased risk of tuberculin skin test conversion [28]. We conducted the largest prospective cohort study of vitamin D and incidence of TB to date among HIV-infected Tanzanian adults initiating ART [15]. In this study we determined individuals with 25(OH)D levels <20 ng/mL at ART initiation had approximately three times the risk of incident pulmonary TB as compared to adults with >30 ng/mL and this relationship persisted after excluding TB cases in the first 2 months of ART to effectively rule out reverse causation [15]. Overall, there is a growing base of observational evidence that HIV-infected and HIV-uninfected individuals with low vitamin D may be at increased risk of developing pulmonary TB.

Randomized controlled trials of vitamin D treatment for individuals with pulmonary TB have produced mixed findings on sputum conversion and other TB treatment outcomes; however, no randomized controlled trials of vitamin D have been conducted for prevention of pulmonary TB disease to the best of our knowledge [30]. One small randomized trial conducted in Mongolia examined the effect of vitamin D_3_ supplementation on tuberculin skin test conversion among 120 children [31]. In this trial children supplemented with 800 IU vitamin D_3_/day had about a 60% reduction in the risk of tuberculin skin test conversion during the 6 months of follow-up as compared to children randomized to placebo, but the results were not statistically significant [31]. A major barrier to conducting pulmonary TB prevention trials in the general population is the large sample size required due to low rates of TB activation (1% of those with positive tuberculin skin test per year) [32]. Nevertheless, these sample size issues are not insurmountable since incident pulmonary TB endpoints are feasible in trials of HIV-infected populations. TB activation rates are 7–10% per year in individuals with advanced HIV disease [8]. As a result, randomized controlled trials of vitamin D supplementation for TB prevention among HIV-infected individuals can be used to inform if larger and more costly trials are needed among HIV-uninfected populations, including TB contacts.

Overall, there is a growing biologic and epidemiologic evidence base which suggests HIV-infected individuals with low vitamin D levels may have increased risk of incident pulmonary TB disease and mortality; however, no randomized controlled trials of vitamin D supplementation have been published to date. In order to provide much needed causal evidence, we present the protocol for an ongoing individually randomized, parallel group, double-blind, placebo-controlled trial of vitamin D_3_ supplementation conducted among HIV-infected individuals initiating ART in Dar es Salaam, Tanzania.

## Methods/Design

**Fig. 1:**
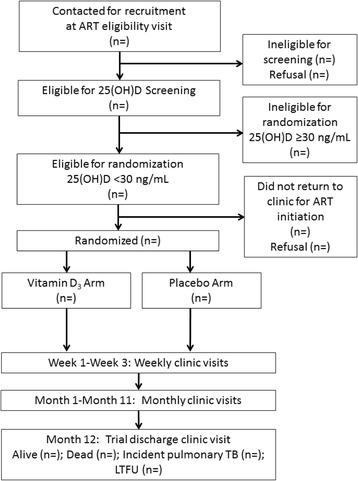
Trial of Vitamins-4 (ToV4) flow diagram. *25(OH)D* 25-hydroxyvitamin D, *ART* antiretroviral therapy, *LTFU* loss to follow-up, *TB* tuberculosis

**Fig. 2:**
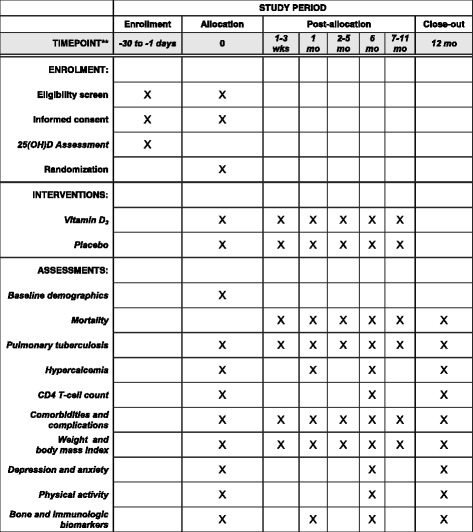
Schedule of enrollment, interventions and assessments (SPIRIT figure). *25(OH)D* 25-hydroxyvitamin D

The Trial of Vitamins-4 (ToV4) is an individually randomized, parallel group, double-blind, placebo-controlled trial of vitamin D_3_ (cholecalciferol) supplementation among HIV-infected adults initiating ART in Dar es Salaam, Tanzania (ClinicalTrials.gov identifier NCT01798680). The trial protocol was developed by collaborators at the Harvard T.H. Chan School of Public Health in the United States and Management and Development for Health (MDH) in the United Republic of Tanzania. The ToV4 protocol diagram is presented in Fig. 1. The schedule of trial enrollment, interventions, and assessments is presented in Fig. 2. The first participant was enrolled in the trial on 24 February 2014 and enrollment is expected to continue until March 2017 with follow-up data collection continuing until March 2018. This protocol was written following the Standard Protocol Items: Recommendations for Interventional trials (SPIRIT) checklist (see Additional file 1).

### Primary and secondary objectives

The overall goal of ToV4 is to investigate a vitamin D_3_ as a simple and low-cost intervention to prolong and improve quality of life for HIV-infected women and men who are initiating ART. The two primary aims of the study are to determine the effect of a vitamin D_3_ supplementation regimen on the hazard of incident (1) mortality and (2) pulmonary TB as compared to a matching placebo regimen during the time period of enrollment at ART initiation to discharge at 1 year of follow-up. The secondary aims of the trial are to examine the effect of the vitamin D_3_ regimen on (a) post-randomization CD4 T cell count as mean difference in cells/μL, (b) incidence of comorbidities and complications associated with HIV and/or ART, (c) post-randomization body mass index (BMI) as mean difference in kg/m^2^, (d) incidence of wasting (BMI <18.5 kg/m^2^) and >10% weight loss from baseline, (e) post-randomization depression and anxiety as mean difference in Hopkins Symptom Checklist (HSCL) scores, (f) post-randomization physical activity as mean difference in metabolic equivalents (METs), (g) post-randomization bone health biomarkers [parathyroid hormone (PTH) and alkaline phosphatase (ALP)] as mean difference in concentrations (h) post-randomization immunologic biomarkers [interlukin-2 (IL-2), interlukin-12 (IL-12), interferon-gamma (IFN-γ), and the antimicrobial peptide cathelicidin] as mean difference in concentrations and (i) risk of hypercalcemia (albumin-adjusted calcium >2.6 mmol/L).

### Study population

Individuals are eligible for the trial randomization if they meet all the following inclusion criteria: (a) adult men or women aged ≥18 years old, (b) HIV-infected, (c) initiating ART at the time of randomization, (d) have low vitamin D levels with a 25(OH)D concentration <30 ng/mL at ART initiation (e) intending to stay in Dar es Salaam for at least 1 year after enrollment, and (f) provide written informed consent. Exclusion criteria include: (a) pregnancy (women of childbearing age are required to have a negative pregnancy test within a month prior to randomization) and (b) enrolled in any other clinical trial. During the trial the ART initiation criteria for the Dar es Salaam HIV treatment program changed from CD4 T cell count <350 cells/μL or WHO HIV stage 3 or 4 disease to CD4 T cell count <500 cells/μL or WHO HIV stage 3 or 4 disease in January 2016.

### Vitamin D_3_ and placebo regimens

The investigational vitamin D_3_ trial regimen consists of oral supplements containing 50,000 IU vitamin D_3_ taken at randomization and once a week for 3 weeks at clinic visits under direct observation (four doses) followed by daily oral supplements containing 2000 IU vitamin D_3_ taken in the home starting at the fourth week until trial discharge at 1 year post ART initiation. The matching placebo regimen consists of oral placebo supplements taken at randomization and once a week for 3 weeks at clinic visits under direct observation (four doses) followed by daily oral placebo supplements taken in the home starting at the fourth week until trial discharge at 1 year post ART initiation.

We decided to use vitamin D_3_, rather than vitamin D_2_ (ergocalciferol), since vitamin D_3_ is more effective in increasing and sustaining levels of circulating 25(OH)D [33]. In addition, the two-stage vitamin D_3_ supplementation regimen was selected in order to quickly and safely boost 25(OH)D levels with the weekly 50,000 IU supplements followed by maintenance of 25(OH)D levels thereafter with the daily 2000 IU supplements. The majority of mortality for HIV-infected individuals initiating ART in resource-limited settings occurs in the first months of treatment and as a result boosting 25(OH)D levels quickly with large doses of vitamin D_3_ may produce a larger effect than daily supplements which would more gradually increase 25(OH)D over time [2]. A randomized trial using a similar two-stage regimen of weekly 50,000 IU vitamin D_3_ for 4 weeks followed by monthly 50,000 IU vitamin D_3_ maintenance doses of vitamin D_3_ among adults with primary hyperparathyroidism found 25(OH)D levels increased 19 ng/mL from baseline to 6 months and maintained levels up to 1 year [34]. We expect a similar approximately 20 ng/mL increase in 25(OH)D from randomization to 6 months. In our trial, we predict nearly all individuals in the vitamin D treatment arm will obtain 25(OH)D levels >30 ng/mL and expect no participant to exceed 85 ng/mL based on the distribution of 25(OH)D in our preliminary observational cohort study [16]. In terms of safety, vitamin D_3_ supplements containing 50,000 IU taken weekly have been found safe and effective in increasing 25(OH)D among individuals with breast cancer, multiple sclerosis, and chronic kidney disease [35–37]. Trials of 2000 IU vitamin D_3_ supplements taken daily have found the regimen maintained or slightly increased 25(OH)D overtime with very low risk of side effects [38–40].

Inclusion of a placebo control was determined to be ethical since it is not known whether the relationship of serum 25(OH)D with morbidity and mortality is causal based on evidence from observational cohort studies and it is also unknown if vitamin D supplementation starting at ART initiation can increase 25(OH)D levels during the relevant biological window to have an effect on mortality, pulmonary TB, and other treatment outcomes [15, 16]. It is also possible vitamin D supplements may have a negative effect by slowing CD4 T cell immune reconstitution due to promotion of Th2 responses, which furthers the principle of equipoise [8, 16]. In addition, a trial of vitamin D supplementation adjunct to TB treatment conducted in Guinea-Bissau found some indication of increased risk of mortality for TB/HIV co-infected participants in the vitamin D3 arm as compared with the placebo arm [41].

The vitamin D_3_ and placebo regimen for ToV4 are produced in the United States by Tischcon Corp. (Salisbury, MD). There is no discernible difference between vitamin D_3_ and placebo supplements in appearance or taste. The regimen bottles for vitamin D_3_ and placebo are also identical except for a unique participant ID label. The weekly 50,000 IU vitamin D_3_ and matching placebo supplements are single hard gelatin capsules containing a light creamy powder which weigh on average 765 mg. The weekly regimen is stored in bottles containing five capsules (one extra supplement), which are pre-labeled with the participant’s study ID. The daily 2000 IU vitamin D_3_ supplements and placebo supplements are hard gelatin capsules containing white powder which weigh on average 477 mg. The daily regimen is stored in bottles containing 35 capsules which are pre-labeled with the patient ID. The vitamin D_3_ and placebo regimens have a shelf-life of 2 years and as a result two batches are required to complete the trial. In order to monitor composition of the supplements throughout their shelf-life, Tishcon Corp. tests a random sample of capsules for the amount of vitamin D_3_ contained in each capsule by high-performance liquid chromatography (HPLC) at production and then again at the end of the shelf-life after return shipping from Tanzania. Table 1 presents the content of vitamin D_3_ and placebo supplements for the first and second regimen batches. The first batch of vitamin D_3_ and placebo supplements contained the expected amount of vitamin D_3_ at production and maintained composition for the duration of the shelf-life.

**Table 1:** Vitamin D_3_ content of trial supplements by batch at production and at end of shelf-life as assessed by high-performance liquid chromatography (HPLC)

	Batch #1 at production (December 2013)	Batch #1 at end of shelf-life (September 2015)	Batch #2 at production (November 2015)	Batch #2 at end of shelf-life
50,000 IU Vitamin D_3_	50,949 IU (101.9%)	48,210 IU (96.4%)	52,325 IU (104.7%)	Currently in use
Matching Placebo for 50,000 IU	None detected	None detected	None detected	Currently in use
2000 IU Vitamin D_3_	2076 IU (103.8%)	2103 IU (105%)	2162 IU (108.1%)	Currently in use
Matching Placebo for 2000 IU	None detected	None detected	None detected	Currently in use

### Sample size

Sample size and power calculations for ToV4 assumed 1:1 randomization to vitamin D_3_ and placebo arms, a nominal Type I error rate (alpha) of 0.05 and a 10% loss to follow-up rate. Due to the possibility of improved care, increased CD4 T cell thresholds for ART initiation and expanded use of isoniazid preventive therapy (IPT) overtime, we selected a trial sample size of 4000 individuals that would provide adequate power for a range of mortality and pulmonary TB rates. Power for the binomial outcomes of mortality and pulmonary TB were calculated based on a two-sided two-sample test for proportions. Assuming a total sample size of 4000 individuals, Table 2 shows the power to detect a range of relative risks for mortality and pulmonary TB for the vitamin D_3_ arm compared with the placebo arm with varying cumulative incidence of mortality and pulmonary TB in the placebo arm. In terms of the mortality endpoint, the ToV4 with an expected sample size of 4000 will have excellent power to detect a relative risk of 0.70 or less (≥90% power), even if the 1-year incidence of mortality in the placebo group decreases to 10% (90% power). A sample size of 4000 will also have 85% power to detect a relative risk of 0.75 if the 1-year mortality rate is 12.5%. This range of detectable effect sizes for mortality is reasonable based on our preliminary observational cohort study which found a 50% increase in risk of mortality among HIV-infected individuals initiating ART with vitamin D deficiency as compared to sufficiency [16]. The cumulative incidence of pulmonary TB during the first year of ART among HIV-infected adults enrolled in ART programs in Africa varies between 5 and 10% [42]. The ToV4 with a sample size of 4000 has excellent power (>85%) to detect relative risk 0.60 or less and has good power (>79%) to detect a relative risk of 0.70 if the incidence of pulmonary TB in the placebo arm is greater than 7.5%. This range of detectable effect sizes for pulmonary TB is also reasonable given our observational study of HIV-infected adults initiating ART determined individuals with vitamin D deficiency had 2.89 (95% CI: 1.31–7.41) times the hazard of incident pulmonary TB as compared to vitamin D sufficient individuals [15].

**Table 2:** Statistical power for a trial sample size of 4000 with 1:1 randomization to vitamin D_3_ and placebo arms, a nominal Type I error rate (alpha) of 0.05 and a 10% loss to follow-up rate

*Mortality*			
	Relative risk of mortality		
Cumulative 1-year incidence mortality in placebo arm	RR = 0.75	RR = 0.70	RR = 0.65
10%	76%	90%	97%
12.5%	85%	95%	99%
15%	91%	98%	99%
*Pulmonary tuberculosis (TB)*			
	Relative risk of pulmonary TB		
Cumulative incidence pulmonary TB in placebo arm	RR = 0.70	RR = 0.65	RR = 0.60
5%	61%	75%	86%
7.5%	79%	90%	97%
10%	90%	97%	99%

### Enrollment and follow-up procedures

#### Screening procedures

The screening visit for ToV4 is integrated with the ART eligibility visit of the Dar es Salaam HIV care and treatment program. At the screening visit, clinic doctors identify HIV-infected adults who are eligible for ART initiation by WHO disease stage or CD4 T cell counts and refer these individuals to research nurses for ToV4 screening procedures. Research nurses assess inclusion/exclusion criteria for the trial and if eligible also seek written informed consent for a blood draw to screen serum 25(OH)D levels. In order to assess pregnancy status of potential participants, all females are asked the date of their last menstrual period and any woman who has not had their menstrual period in the previous 6 weeks is given a pregnancy test. Individuals who meet all eligibility for ToV4 screening and consent for a blood draw have 5 mL of blood collected in a red-top vacutainer tube for serum 25(OH)D assessment. At the screening visit, all participants receive treatment of comorbidities per Tanzanian standard of care and are scheduled for an ART initiation visit in 1–2 weeks in concordance with the HIV treatment program visit protocol.

#### Screening 25(OH)D assessment

**Fig. 3:**
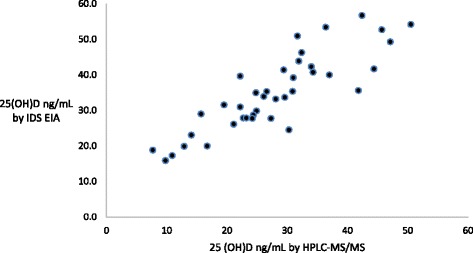
Scatterplot of validation study results comparing 25(OH)D concentration assessed by IDS EIA (y-axis) versus HPLC-MS/MS (x-axis). *25(OH)D* 25-hydroxyvitamin D, *EIA* enzyme immunoassay, *HPLC-MS/MS* high-performance liquid chromatography tandem mass spectrometry, *IDS* Immunodiagnostics

Blood samples collected at the screening visit are then sent to a central laboratory where sera are separated by centrifugation. 25(OH)D concentration was then manually assessed with the commercial Immunodiagnostics (IDS) enzyme immunoassay (EIA) per kit protocols (IDS, Boldon, UK). Prior to the start of the trial we conducted a validation study of the IDS EIA using sera from our previous observational cohort of HIV-infected Tanzanian men and women initiating ART due to reports of overestimation of serum 25(OH)D by EIA [16, 43]. The validation study was conducted at the Boston Children’s Hospital Laboratory in Boston, MA, USA. In the validation study 40 randomly selected sera had 25(OH)D assessed both by the IDS EIA and by high-performance liquid chromatography tandem mass spectrometry (HPLC-MS/MS) using an API-5000 (AB Sciex, Foster City, CA, USA) as the ‘gold standard’ [44]. The IDS EIA was manually performed per commercial kit instructions. For HPLC-MS/MS testing, serum samples were first extracted and ‘cleaned up’ using the Aria-TLX-2 (Thermo Fisher Scientific, Waltham, MA, USA) after which 50 μL was mixed with acetonitrile containing internal standard of 25-hydroxyvitamin d6D3. Samples were then centrifuged and 50 μL of the supernatant was injected into the Aria-TLX-2, passed through a Cyclone-P column (Thermo Fisher Scientific), and then eluted through a Kinetex C column (Phenomenex, Torrance, CA, USA). The eluate was injected into the API-5000 for atmospheric pressure chemical ionization and passed through the triple quadrupole mass spectrometer for detection and quantified measurements. This HPLC-MS/MS method is linear up to 100 ng/mL and sensitive to 1 ng/mL. Figure 3 presents a scatterplot of the validation study results, which show the IDS EIA linearly overestimated 25(OH)D concentration as compared with the HPLC-MS/MS. Robust regression determined the line of best fit was IDS EIA 25(OH)D ng/mL = 10.451+ 0.886*HPLC-MS/MS 25(OH)D ng/mL. Accordingly, in order to enroll individuals with 25(OH)D <30 ng/mL we set the threshold for trial eligibility with the IDS EIA test kit to 25(OH)D <37 ng/mL.

#### Randomization procedures

Individuals screened for 25(OH)D levels return to the clinic within 1–2 weeks for the trial randomization visit which is integrated with the ART initiation visit of the HIV treatment program. At the randomization visit, research nurses inform all screened individuals of their 25(OH)D results. Individuals with 25(OH)D levels ≥30 ng/mL are told they have adequate levels of vitamin D and are not eligible for trial enrollment. All individuals with 25(OH)D levels <30 ng/mL are informed they have low vitamin D levels as well as potential sources of vitamin D (sun exposure, oily fish, eggs, etc.). Research nurses then re-document all eligibility criteria for individuals with 25(OH)D <30 ng/mL and seek written informed consent for ToV4 enrollment.

Individuals who consent for ToV4 enrollment are individually randomized 1:1 to the vitamin D_3_ or placebo arm. Randomization procedures were designed for complete allocation concealment and blinding of all participants, clinic staff, senior research staff, and trial statisticians. Trial arm allocation is performed according to a computer-generated randomization list of 4000 individuals with sequence blocks of ten stratified by study clinic that was generated by a non-study statistician. Two non-study statisticians will hold the randomization list codes until completion of primary analyses or until requested by the Data and Safety Monitoring Board (DSMB). An independent study pharmacist prepares the vitamin D_3_ and placebo regimen by labeling bottles with participant IDs based on the randomization list. At the randomization visit, allocation concealment is performed by assigning individuals eligible for trial randomization to the next available numeric participant ID which corresponds to a set of pre-labeled regimen bottles with the same participant ID. As a result, all trial participants, research staff, and trial statisticians are not able to determine the allocated trial arm for any trial participant or identify trial participants who are on the same trial arm.

At the randomization visit, all study participants receive a full clinical examination from study physicians, and have HIV disease stage assessed according to the WHO criteria [45]. All comorbidities are treated per Tanzanian standard of care. Nurses also administer a standardized questionnaire to collect information on sociodemographics, reported symptoms during the last 30 days (fever, cough, etc), depression, anxiety, social support, and physical activity. Nurses also collect anthropometric measurements including height, weight, and mid-upper arm circumference. Participants are also asked for cell phone numbers and location of their home for follow-up visits, given the participant provides written informed consent for phone and home contact with study staff. At the end of the randomization visit, research nurses directly observe the participant taking the first supplement of the weekly regimen. The weekly regimen bottles remain in the clinic for all participants.

#### Follow-up clinic visit procedures

All participants are actively followed at weekly clinic visits for 3 weeks (week 1, 2, 3) followed by monthly clinic visits at starting at the fourth week until 12 months post ART initiation. At 12 months, participants are discharged from the trial and continue to receive standard treatment within the HIV care and treatment program but are not followed by research staff. At all clinic visits research nurses administer a questionnaire on symptoms reported within the last 30 days and collect anthropometric measurements including weight and mid-upper arm circumference. At the 6-month and 12-month visit the baseline questionnaire on sociodemographic, depression, anxiety, social support, and physical activity is re-administered. Physicians also perform a full clinical examination, assess WHO HIV disease stage, and treat all comorbidities in accordance to Tanzanian standard of care at all study visits. At week 1, week 2, and week 3 clinic visits, nurses directly observe the participant taking the trial supplement in study clinics. At the fourth week and at monthly visits thereafter until trial discharge at 12 months, participants are given regimen bottles containing 35 trial supplements to be taken daily in the home.

### Primary and secondary outcome assessment

#### Vital status assessment

Participants who do not come for their scheduled clinic appointments are contacted via cell phone and/or visited at home to assess vital status. Participants that are reached by phone or in the home are asked to come to the clinic if the participant is physically able to do so. For patients who traveled out of Dar es Salaam, we maintain contact via cell phone or with neighbors and relatives to collect information on the vital status of the participant. For participants who are confirmed to have died we administer a standardized verbal autopsy to relatives or individuals present around the time of death to assign a cause of death. Participants that die in a health facility also have events before and at the time of death transcribed from hospital records. Two seniors HIV clinicians utilize the verbal autopsy data and hospital records to establish a cause of death by consensus. A third senior clinic serves as the tie-breaker for deaths for which the first two clinicians cannot reach consensus.

#### Pulmonary TB assessment

All participants presenting with any of the following five clinical signs and symptoms at any study visit are considered pulmonary TB suspects per the Ministry of Health of Tanzania protocol: cough for 2 weeks or more, fever, 3 kg weight loss in 1 month or noticeable weight loss for new patients, night sweats, or hemoptysis. All TB suspects provide a spot sputum specimen at the clinic visit symptoms were noted and are given a sputum container for collection of early morning sputum before a second visit the next weekday. A third spot specimen is then collected under supervision by a nurse at the second clinic visit. The sputum smears are prepared and stained using Ziehl-Nielsen technique and examined for acid-fast bacilli (AFB) by trained health laboratory technologists who grade the smears on an ordinal scale following standard guidelines of the International Union Against Tuberculosis and Lung disease [46]. All TB suspects also receive a chest X-ray. During the trial the GeneXpert System (Cepheid, Sunnyvale, CA, USA) which uses the cartridge-based Xpert MTB/RIF (Xpert) assay became available for TB testing at three of the four study clinics. Participants who are sputum AFB negative for whom TB is still suspected are tested by GeneXpert for detection of Mtb. DNA. According to Tanzanian national guidelines, pulmonary TB diagnosis is made when at least one sputum smear is positive for AFB, GeneXpert detects Mtb DNA, or when there are radiological features suggestive of TB in the presence of constitutional TB symptoms. Patients start TB treatment on same the day of diagnosis.

#### Hypercalcemia assessment

All participants have serum calcium and albumin assessed at baseline, 1, 6 and 12 months. Serum calcium and albumin concentrations are assessed with the Roche Cobas Integra 400 Plus (Roche Diagnostics, Basel, Switzerland). Albumin-adjusted serum calcium concentrations are then calculated using the following equation: calcium (mg/dL) = total calcium (mg/dL) + 0.8 × [4 − albumin (g/dL)] [47]. Any participants with above the normal physiologic range of albumin-adjusted calcium (>2.6 mmol/L) are diagnosed with hypercalcemia, discontinue their trial regimen for the duration of the trial and are given appropriate clinical management. Individuals with hypercalcemia continue to be followed up until discharge of the trial and resolution of hypercalcemia. In addition participants are monitored at all clinic visits by study physicians for symptoms of hypercalcemia including: nausea, vomiting, excessive thirst, anorexia, symptoms of kidney stones, and confusion. If any of these symptoms are reported and there is no other suspected cause, study clinicians order calcium and albumin testing. At this point participants with suspected hypercalcemia stop their randomized treatment regimen until calcium testing results are returned. If albumin-adjusted calcium levels are normal for hypercalcemia suspects, the patient is allowed to re-start the regimen under discretion of study physicians. Hypercalcemia suspects with albumin-adjusted calcium >2.6 mmol/L discontinue their trial regimen for duration of the trial and are given appropriate clinical management.

#### Laboratory investigations

All participants have CD4 T cell counts assessed at or before the ToV4 screening/ART eligibility visit and then every 6 months after ART initiation. Hemoglobin is also measured at the ToV4 screening/ART eligibility visit. In order to examine the effect of the vitamin D_3_ regimen on bone health, we will measure PTH and ALP at baseline, 1, 6, and 12 months for a random sample of 800 individuals (20%). We will also assess the effect of vitamin D_3_ on host immune response through measurement of IL-2, IL-12, IFN-γ, and the antimicrobial peptide cathelicidin in 800 (20%) randomly selected individuals at baseline, 1, 6, and 12 months.

#### Compliance assessment

Any trial requires a high degree of compliance with experimental regimens as non-compliance can bias results and decrease the statistical power of a study. The best way to ensure full compliance is to observe each participant ingesting treatment. In ToV4 research nurses directly monitor subjects taking the weekly trial supplements (randomization, week 1, week 2, week 3), but this type of monitoring is not practical for the subsequent daily supplements taken in the home (week 4 to month 12). In order to promote adherence during the daily regimen period, all participants are encouraged to use the daily supplements at the same time every day, to put the bottle in a visible place, and identify an adherence assistant who would remind her/him to take the supplement. Compliance with the daily ToV4 supplements will be assessed three ways: (a) direct questioning about use of the supplements in the previous 24 hours and 2 weeks, (b) pill count from returned daily regimen bottles, and (c) biochemical assessment of 25(OH)D in a subset of participants longitudinally at randomization, 1, 6, and 12 months.

#### Standard of care

All study participants are provided with HIV care and treatment that adhere to Tanzanian national guidelines. During the trial the ART initiation criteria within the Dar es Salaam HIV treatment program changed from CD4 T cell count <350 cells/μL or WHO HIV stage 3 or 4 disease to CD4 T cell count <500 cells/μL or WHO HIV stage 3 or 4 disease in January 2016. Per Tanzanian guidelines, zidovudine, lamivudine, and efavirenz are the choice first-line regimen with tenofovir, nevirapine, and stavudine as back-up drugs. The standard visit schedule for patients on ART is clinic visits at 2 weeks and 1 month post-initiation with monthly thereafter. Patients who are stable after 6 months of ART are eligible receive a 2-month supply of ART with a subsequent scheduled clinic visit at corresponding 2-month intervals. CD4 T cell counts are assessed at the ART eligibility visit and every 6 months after ART initiation. During the trial HIV viral load testing became available as standard of care with assessment every 6 months after ART initiation. Prophylaxis for opportunistic infections is provided in line with the Tanzanian HIV treatment guidelines. Participants receive co-trimoxazole prophylaxis if CD4 cell count is <200 cells/μL. All participants diagnosed with TB are treated according to the WHO-approved guidelines of the National Tuberculosis and Leprosy Program (NTLP) in Tanzania. All TB patients receive directly observed therapy (DOT) with a 6-month short course regimen consisting of a 2-month intensive phase of daily rifampicin/isoniazid/pyrazinamide/ethambutol (RHZE) combination tablets, followed by a 4-month continuation phase of daily rifampicin and isoniazid (RH) combination tablets. Sputa from smear-positive TB patients is collected and examined at 0, 2 and 5 months and at any other time if recurrence of TB is suspected. If the direct sputum smear is negative at the end of 2 months, the continuation phase is started and if a smear is still positive at 3 months the patient is checked for multi-drug resistance. Treatment failures and relapses are treated with an 8-month re-treatment regimen containing rifampicin throughout, with the intensive period with RHZE and streptomycin injection continuing for 3 months. Psychological and nutritional support is also provided as standard of care.

### Statistical analysis plan

An intent-to-treat analysis will be used as the primary analytic strategy for all analyses. The primary efficacy trial endpoints for this trial are (1) death and (2) incident pulmonary TB. The primary safety endpoint is hypercalcemia (albumin-adjusted calcium >2.6 mmol/L). We will use the use the log-rank test to test the differences in the incidence rates of death and pulmonary TB between the treatment arms, and use the Fisher’s exact test to test differences in proportion of participants with incident hypercalcemia.

The secondary endpoints for the study include: (a) post-randomization CD4 T cell count, (b) incidence of comorbidities and complications associated with HIV and/or ART, (c) post-randomization BMI, (d) incidence of wasting (BMI <18.5 kg/m^2^) and >10% weight loss from baseline, (e) post-randomization depression and anxiety scores as assessed by the Hopkins Symptom Checklist (HSCL), (f) post-randomization physical activity as measured by METs, (g) post-randomization bone health biomarker levels (PTH and ALP) and (h) post-randomization immunologic biomarker levels (IL-2, IL-12, IFN-γ, and cathelicidin). We will use linear mixed-effects models with a random intercept, a compound symmetric covariance structure, and robust standard errors to assess mean differences between baseline and post-randomization levels of CD4 T cell count, BMI, METs, 25(OH)D, PTH, ALP, IL-2, IL-12, IFN-γ, and cathelicidin by randomization group. Linear mixed-effects models will lead to robust inference for the fixed effects even if the covariance structure is misspecified. Linear mixed-effects models will also account for within-subject correlation, and accommodate varying number and timing of measurements per participant over time. We will use a generalized linear mixed model with random intercept, a compound symmetric covariance structure, and robust standard errors to assess differences in the incidence of comorbidities and complications associated with HIV and/or ART which are repeatable binomial events. The log-rank test will be used to assess differences in the incidence rates of wasting and >10% weight loss between the treatment arms.

We will also examine effect modification of any treatment effect by pre-defined baseline variables: 25(OH)D concentration, sex, age, BMI, CD4 T cell count, hemoglobin concentration, WHO HIV disease stage, pulmonary TB, receipt of isoniazid preventive therapy, ART regimen, and trial regimen compliance. To assess the statistical significance of each interaction, we will use the likelihood ratio test for risk-ratio homogeneity in the mortality, pulmonary TB, and hypercalcemia analyses and the score test in the linear mixed-effects and generalized linear mixed models for longitudinal secondary outcomes.

## Discussion

Antiretroviral therapy coverage is rapidly expanding globally and treatment programs are in need of interventions to prolong and improve the quality of life for HIV-infected individuals in resource-limited settings, especially during the initial months of treatment [2, 3]. Vitamin D_3_ supplementation may be an effective ART adjunct intervention since vitamin D deficiency is common among HIV-infected individuals, ART can further reduce vitamin D levels by altering vitamin D metabolism, multiple observational cohort studies have determined low vitamin D levels are associated with increased risk of mortality, pulmonary TB, and disease progression among HIV-infected individuals, and vitamin D_3_ supplements are known to be effective and safe in improving vitamin D status [6, 8, 11, 15, 16, 34, 37]. Based on this rationale, the ToV4 was designed to establish the causal effect vitamin D_3_ supplementation on mortality and incidence of pulmonary TB among HIV-infected adults initiating ART in Dar es Salaam, Tanzania. A search of clinicaltrials.gov determined the only other clinical trial of vitamin D_3_ among HIV-infected individuals with mortality or morbidity outcomes listed as primary or secondary outcomes is an ongoing study called the “Trial of Vitamin D in HIV Progression, Birth Outcomes, and Child Health” (NCT02305927). This ongoing trial is also conducted by our research group and is a randomized, double-blind, placebo-controlled trial of vitamin D_3_ supplementation among HIV-infected pregnant women in Dar es Salaam, Tanzania. Accordingly, the ToV4 will likely be the first randomized trial to determine the effect of vitamin D_3_ supplementation on mortality and morbidity outcomes among HIV-infected adult men and non-pregnant women.

The study population of the ToV4 was purposely defined by inclusion and exclusion criteria to select a population that may have significant benefit from vitamin D_3_ supplementation but also allow for generalizability of the findings. In the trial we only enroll HIV-infected adults initiating ART with 25(OH)D levels <30 ng/mL and exclude individuals with ≥30 ng/mL from randomization procedures. The 25(OH)D inclusion criteria was based on our observational cohort study of HIV-infected adults initiating ART in Tanzania which suggested there is likely little to no benefit to obtaining 25(OH)D levels >30 ng/mL in terms of mortality and pulmonary TB in our study population [15, 16]. The restriction of the trial population to individuals <30 ng/mL 25(OH)D also assists with translation of the findings as HIV programs in similar settings will be able estimate the potential impact of implementing vitamin D_3_ supplementation based on their local prevalence of 25(OH)D <30 ng/mL. We also tried to maintain the generalizability of our findings by defining the study population as adults initiating ART broadly and did not restrict the study population by CD4 T cell and WHO disease stage criteria. HIV treatment programs in Tanzania and sub-Saharan Africa are moving from using a <350 to a <500 CD4 T cells/μL threshold for ART initiation based on WHO guidelines [48]. As we expected, the Dar es Salaam HIV treatment program changed ART initiation guidelines from <350 to a <500 CD4 T cells/μL threshold to a ‘test-and-treat’ strategy during the trial and as a result the general ToV4 population of adults initiating ART became on average healthier over the course of the trial as the new guideline were gradually implemented. Nevertheless, the slight decrease in power due to lower event rates is outweighed by the need for our trial to reflect the population of adults initiating ART for possible implementation of vitamin D_3_ supplementation in the treatment program. The ToV4 DSMB reviews event rates every 6 months and will recommend any necessary changes to the trial sample size to maintain statistical power. Overall, the ToV4 study population was carefully designed to include individuals who may experience the greatest benefits of vitamin D_3_ supplementation, but will also preserve the generalizability and potential ability to translate the findings into practice in Dar es Salaam and similar HIV treatment programs.

Overall, the ToV4 will provide causal evidence if vitamin D_3_ should be considered as an ART adjunct intervention to reduce pulmonary TB and mortality among HIV-infected adults initiating treatment. The trial will also provide evidence for the effect of vitamin D_3_ supplementation on a wide range of secondary outcomes including: CD4 T cell reconstitution, non-pulmonary TB comorbidities and complications associated with HIV and/or ART, bone health, immunologic biomarkers, weight gain, depression and anxiety, physical activity, and incidence of hypercalcemia. The results of the trial are likely generalizable to HIV-infected adults with low vitamin D who are initiating ART in similar resource-limited settings and the trial findings will also inform whether larger vitamin D_3_ supplementation trials for the prevention of TB in HIV-uninfected populations should be pursued.

### Trial status

Trial enrollment started on 24 February 2014 and recruitment is ongoing as of 26 January 2017.

## Additional file

Additional file 1: SPIRIT 2013 checklist: recommended items to address in a clinical trial protocol and related documents (DOC 122 kb)

## Abbreviations

25(OH)D: 25-hydroxyvitamin D
AFB: Acid-fast bacilli
ALP: Alkaline phosphatase
ART: Antiretroviral therapy
BMI: Body mass index
EIA: Enzyme immunoassay
HIV: Human immunodeficiency virus
HPLC-MS/MS: High-performance liquid chromatography tandem mass spectrometry
IDS: Immunodiagnostics
IFN: Interferon
IL: Interleukin
MDH: Management and Development for Health
METs: Metabolic equivalents
Mtb: *Mycobacterium tuberculosis*
NTLP: National Tuberculosis and Leprosy Program
PTH: Parathyroid hormone
RHZE: Rifampicin/isoniazid/pyrazinamide/ethambutol
TB: Tuberculosis
ToV4: Trial of Vitamins-4
WHO: World Health Organization

## References

[1] Joint United Nations Programme on HIV/AIDS (2016). Global AIDS update 2016.

[2] Braitstein P, Brinkhof MW, Dabis F, Schechter M, Boulle A, Miotti P, Wood R, Laurent C, Sprinz E, Seyler C (2006). Mortality of HIV-1-infected patients in the first year of antiretroviral therapy: comparison between low-income and high-income countries. Lancet.

[3] Brinkhof MW, Boulle A, Weigel R, Messou E, Mathers C, Orrell C, Dabis F, Pascoe M, Egger M, International Epidemiological Databases to Evaluate A (2009). Mortality of HIV-infected patients starting antiretroviral therapy in sub-Saharan Africa: comparison with HIV-unrelated mortality. PLoS Med.

[4] Chalamilla G, Hawkins C, Okuma J, Spiegelman D, Aveika A, Christian B, Koda H, Kaaya S, Mtasiwa D, Fawzi W (2012). Mortality and treatment failure among HIV-infected adults in Dar Es Salaam, Tanzania. J Int Assoc Physicians AIDS Care (Chic).

[5] Fabri M, Stenger S, Shin DM, Yuk JM, Liu PT, Realegeno S, Lee HM, Krutzik SR, Schenk M, Sieling PA (2011). Vitamin D is required for IFN-gamma-mediated antimicrobial activity of human macrophages. Sci Transl Med.

[6] Deluca HF, Cantorna MT (2001). Vitamin D: its role and uses in immunology. FASEB J.

[7] Bar-Shavit Z, Noff D, Edelstein S, Meyer M, Shibolet S, Goldman R (1981). 1,25-dihydroxyvitamin D3 and the regulation of macrophage function. Calcif Tissue Int.

[8] Villamor E (2006). A potential role for vitamin D on HIV infection?. Nutr Rev.

[9] Haug C, Muller F, Aukrust P, Froland SS (1994). Subnormal serum concentration of 1,25-vitamin D in human immunodeficiency virus infection: correlation with degree of immune deficiency and survival. J Infect Dis.

[10] Vescini F, Cozzi-Lepri A, Borderi M, Re MC, Maggiolo F, De Luca A, Cassola G, Vullo V, Carosi G, Antinori A (2011). Prevalence of hypovitaminosis D and factors associated with vitamin D deficiency and morbidity among HIV-infected patients enrolled in a large Italian cohort. J Acquir Immune Defic Syndr.

[11] Viard JP, Souberbielle JC, Kirk O, Reekie J, Knysz B, Losso M, Gatell J, Pedersen C, Bogner JR, Lundgren JD (2011). Vitamin D and clinical disease progression in HIV infection: results from the EuroSIDA study. AIDS.

[12] Shepherd L, Souberbielle JC, Bastard JP, Fellahi S, Capeau J, Reekie J, Reiss P, Blaxhult A, Bickel M, Leen C (2014). Prognostic value of vitamin D level for all-cause mortality, and association with inflammatory markers, in HIV-infected persons. J Infect Dis.

[13] Mehta S, Giovannucci E, Mugusi FM, Spiegelman D, Aboud S, Hertzmark E, Msamanga GI, Hunter D, Fawzi WW (2010). Vitamin D status of HIV-infected women and its association with HIV disease progression, anemia, and mortality. PLoS One.

[14] Ezeamama AE, Guwatudde D, Wang M, Bagenda D, Kyeyune R, Sudfeld C, Manabe YC, Fawzi WW (2016). Vitamin-D deficiency impairs CD4 + T-cell count recovery rate in HIV-positive adults on highly active antiretroviral therapy: a longitudinal study. Clin Nutr.

[15] Sudfeld CR, Giovannucci EL, Isanaka S, Aboud S, Mugusi FM, Wang M, Chalamilla G, Fawzi WW (2013). Vitamin D status and incidence of pulmonary tuberculosis, opportunistic infections, and wasting among HIV-infected Tanzanian adults initiating antiretroviral therapy. J Infect Dis.

[16] Sudfeld CR, Wang M, Aboud S, Giovannucci EL, Mugusi FM, Fawzi WW (2012). Vitamin D and HIV progression among Tanzanian adults initiating antiretroviral therapy. PLoS One.

[17] Havers F, Smeaton L, Gupte N, Detrick B, Bollinger RC, Hakim J, Kumarasamy N, Andrade A, Christian P, Lama JR (2014). 25-Hydroxyvitamin D insufficiency and deficiency is associated with HIV disease progression and virological failure post-antiretroviral therapy initiation in diverse multinational settings. J Infect Dis.

[18] Rook GA, Steele J, Fraher L, Barker S, Karmali R, O'Riordan J, Stanford J (1986). Vitamin D3, gamma interferon, and control of proliferation of Mycobacterium tuberculosis by human monocytes. Immunology.

[19] Liu PT, Stenger S, Li H, Wenzel L, Tan BH, Krutzik SR, Ochoa MT, Schauber J, Wu K, Meinken C (2006). Toll-like receptor triggering of a vitamin D-mediated human antimicrobial response. Science.

[20] Gombart AF, Borregaard N, Koeffler HP (2005). Human cathelicidin antimicrobial peptide (CAMP) gene is a direct target of the vitamin D receptor and is strongly up-regulated in myeloid cells by 1,25-dihydroxyvitamin D3. FASEB J.

[21] Wang TT, Nestel FP, Bourdeau V, Nagai Y, Wang Q, Liao J, Tavera-Mendoza L, Lin R, Hanrahan JW, Mader S (2004). Cutting edge: 1,25-dihydroxyvitamin D3 is a direct inducer of antimicrobial peptide gene expression. J Immunol.

[22] Gibney KB, MacGregor L, Leder K, Torresi J, Marshall C, Ebeling PR, Biggs BA (2008). Vitamin D deficiency is associated with tuberculosis and latent tuberculosis infection in immigrants from sub-Saharan Africa. Clin Infect Dis.

[23] Ho-Pham LT, Nguyen ND, Nguyen TT, Nguyen DH, Bui PK, Nguyen VN, Nguyen TV (2010). Association between vitamin D insufficiency and tuberculosis in a Vietnamese population. BMC Infect Dis.

[24] Grange JM, Davies PD, Brown RC, Woodhead JS, Kardjito T (1985). A study of vitamin D levels in Indonesian patients with untreated pulmonary tuberculosis. Tubercle.

[25] Wilkinson RJ, Llewelyn M, Toossi Z, Patel P, Pasvol G, Lalvani A, Wright D, Latif M, Davidson RN (2000). Influence of vitamin D deficiency and vitamin D receptor polymorphisms on tuberculosis among Gujarati Asians in west London: a case-control study. Lancet.

[26] Martineau AR, Leandro AC, Anderson ST, Newton SM, Wilkinson KA, Nicol MP, Pienaar SM, Skolimowska KH, Rocha MA, Rolla VC (2010). Association between Gc genotype and susceptibility to TB is dependent on vitamin D status. Eur Respir J.

[27] Martineau AR, Nhamoyebonde S, Oni T, Rangaka MX, Marais S, Bangani N, Tsekela R, Bashe L, de Azevedo V, Caldwell J (2011). Reciprocal seasonal variation in vitamin D status and tuberculosis notifications in Cape Town, South Africa. Proc Natl Acad Sci U S A.

[28] Arnedo-Pena A, Juan-Cerdan JV, Romeu-Garcia MA, Garcia-Ferrer D, Holguin-Gomez R, Iborra-Millet J, Pardo-Serrano F (2015). Vitamin D status and incidence of tuberculosis infection conversion in contacts of pulmonary tuberculosis patients: a prospective cohort study. Epidemiol Infect.

[29] Talat N, Perry S, Parsonnet J, Dawood G, Hussain R (2010). Vitamin d deficiency and tuberculosis progression. Emerg Infect Dis.

[30] Kearns MD, Alvarez JA, Seidel N, Tangpricha V (2015). Impact of vitamin D on infectious disease. Am J Med Sci.

[31] Ganmaa D, Giovannucci E, Bloom BR, Fawzi W, Burr W, Batbaatar D, Sumberzul N, Holick MF, Willett WC (2012). Vitamin D, tuberculin skin test conversion, and latent tuberculosis in Mongolian school-age children: a randomized, double-blind, placebo-controlled feasibility trial. Am J Clin Nutr.

[32] Vieth R (2011). Vitamin D, nutrient to treat TB begs the prevention question. Lancet.

[33] Armas LA, Hollis BW, Heaney RP (2004). Vitamin D2 is much less effective than vitamin D3 in humans. J Clin Endocrinol Metabol.

[34] Pepper KJ, Judd SE, Nanes MS, Tangpricha V (2009). Evaluation of vitamin D repletion regimens to correct vitamin D status in adults. Endocr Pract.

[35] Chandra P, Binongo JN, Ziegler TR, Schlanger LE, Wang W, Someren JT, Tangpricha V (2008). Cholecalciferol (vitamin D3) therapy and vitamin D insufficiency in patients with chronic kidney disease: a randomized controlled pilot study. Endocr Pract.

[36] Hiremath GS, Cettomai D, Baynes M, Ratchford JN, Newsome S, Harrison D, Kerr D, Greenberg BM, Calabresi PA (2009). Vitamin D status and effect of low-dose cholecalciferol and high-dose ergocalciferol supplementation in multiple sclerosis. Mult Scler.

[37] Khan QJ, Reddy PS, Kimler BF, Sharma P, Baxa SE, O'Dea AP, Klemp JR, Fabian CJ (2010). Effect of vitamin D supplementation on serum 25-hydroxy vitamin D levels, joint pain, and fatigue in women starting adjuvant letrozole treatment for breast cancer. Breast Cancer Res Treat.

[38] Dong Y, Stallmann-Jorgensen IS, Pollock NK, Harris RA, Keeton D, Huang Y, Li K, Bassali R, Guo DH, Thomas J (2010). A 16-week randomized clinical trial of 2000 international units daily vitamin D3 supplementation in black youth: 25-hydroxyvitamin D, adiposity, and arterial stiffness. J Clin Endocrinol Metab.

[39] Schleithoff SS, Zittermann A, Tenderich G, Berthold HK, Stehle P, Koerfer R (2006). Vitamin D supplementation improves cytokine profiles in patients with congestive heart failure: a double-blind, randomized, placebo-controlled trial. Am J Clin Nutr.

[40] Abu-Mouch S, Fireman Z, Jarchovsky J, Zeina AR, Assy N (2011). Vitamin D supplementation improves sustained virologic response in chronic hepatitis C (genotype 1)-naive patients. World J Gastroenterol.

[41] Wejse C, Gomes VF, Rabna P, Gustafson P, Aaby P, Lisse IM, Andersen PL, Glerup H, Sodemann M (2009). Vitamin D as supplementary treatment for tuberculosis: a double-blind, randomized, placebo-controlled trial. Am J Respir Crit Care Med.

[42] Nicholas S, Sabapathy K, Ferreyra C, Varaine F, Pujades-Rodriguez M, Frontieres AWGoMS (2011). Incidence of tuberculosis in HIV-infected patients before and after starting combined antiretroviral therapy in 8 sub-Saharan African HIV programs. J Acquir Immune Defic Syndr.

[43] Cavalier E, Huberty V, Cormier C, Souberbielle JC (2011). Overestimation of the 25(OH)D serum concentration with the automated IDS EIA kit. J Bone Miner Res.

[44] Lensmeyer GL, Wiebe DA, Binkley N, Drezner MK (2006). HPLC method for 25-hydroxyvitamin D measurement: comparison with contemporary assays. Clin Chem.

[45] World Health Organization (2010). Antiretroviral therapy for HIV infection in adults and adolescents: recommendations for a public health approach-2010 revision.

[46] Enarson DA, Rieder HL, Arnadottir T (1994). Tuberculosis guide for low income countries.

[47] Portale AA (1999). Blood calcium, phosphorus, and magnesium. Primer Metabol Bone Dis Disord Miner Metabol.

[48] World Health Organization (2013). Consolidated guidelines on general HIV care and the use of antiretroviral drugs for treating and preventing HIV infection: recommendations for a public health approach.

